# Treatment of a Double Cancer Patient With Primary Inferior Vena Cava Sarcoma and Lung Adenocarcinoma: A Case Report and Literature Review

**DOI:** 10.3389/fsurg.2022.852757

**Published:** 2022-04-06

**Authors:** Xiaohu Guo, Zhengang Wei, Mancai Wang, Youcheng Zhang

**Affiliations:** Second Department of the General Surgery of Lanzhou University Second Hospital, Lanzhou, China

**Keywords:** sarcoma, inferior vena cava, lung adenocarcinoma, leiomyosarcoma, case report

## Abstract

**Background:**

Leiomyosarcoma of the inferior vena cava (IVC) is a rare malignancy. Here, we present the case of a 38-year-old woman with a primary IVC leiomyosarcoma and lung adenocarcinoma.

**Case Report:**

The patient, a 38-year-old Chinese female, presented to the general surgical outpatients clinic with a 18-month history of intermittent right upper abdominal pain. Contrast-enhanced computed tomography (CT) showed a tumor of IVC (3.4^*^2.7 cm) extending to the renal veins. In addition, chest CT revealed a mass lesion in the upper left lung lobe. Then, the patient underwent resection of the IVC tumor and wedge resection of the upper lobe of the left lung. The patient then received gefitinib (250 mg/day) as a maintenance therapy until the tumor recurrence or metastasis in the follow-up period. Pulmonary metastasis of the sarcoma were first diagnosed 20 month after the resection of the IVC leiomyosarcoma. So the patient again received thoracoscopic wedge pneumonectomy, and it was confirmed to be metastasis of IVC leiomyosarcoma. The patient received oral anlotinib treatment (12 mg once daily) after the last operation. During on-going regular follow-up visits no evidence of recurrence or metastasis was observed from December 2020 to October 2021.

**Conclusions:**

The patient with a primary IVC leiomyosarcoma and lung adenocarcinoma is extremely rare. Surgery is still an effective treatment for patients with a primary IVC leiomyosarcoma and lung adenocarcinoma at present.

## Background

Leiomyosarcoma originating in the inferior vena cava (IVC) is a very rare malignancy, with a few hundred cases reported worldwide (1, 2). They occur more commonly in females and the peak incidence is in the six to seventh decade of life (3). It is difficult to diagnose because of patients often present with non-specific symptoms such as dyspnoea, abdominal pain, distention, back pain, weight loss, and malaise. Primary leiomyosarcoma of the IVC may be under-detected for several years before the diagnosis is made. Because of the lack of early diagnosis and effective treatment, the prognosis of primary IVC leiomyosarcoma is very poor, with a median survival of 2 years post diagnosis (4). Lung cancer has been one of the leading causes of cancer-related death worldwide for two decades (5). Among patients with lung adenocarcinoma, females were more than males. We conducted a search for the case of patient with a primary IVC leiomyosarcoma and lung adenocarcinom in PubMed, Medline and Google Scholar databases and no similar cases were reported. Here, we present the case of a 38-year-old woman with a primary IVC leiomyosarcoma and lung adenocarcinoma. To our knowledge, this is the first report the case of patient with a primary IVC leiomyosarcoma and lung adenocarcinom.

## Case Report

The patient, a 38-year-old Chinese female, presented to the general surgical outpatients clinic with a 18-month history of intermittent right upper abdominal pain, back pain, nausea and vomiting. The examination of the patient's medical history revealed good general health, absence of systemic diseases, and smoking habit. The physical examination was normal. These symptoms were not decreased in patient treated with oral acid-inhibitory drug and cholagogue at the local hospital.

**Figure 1:**
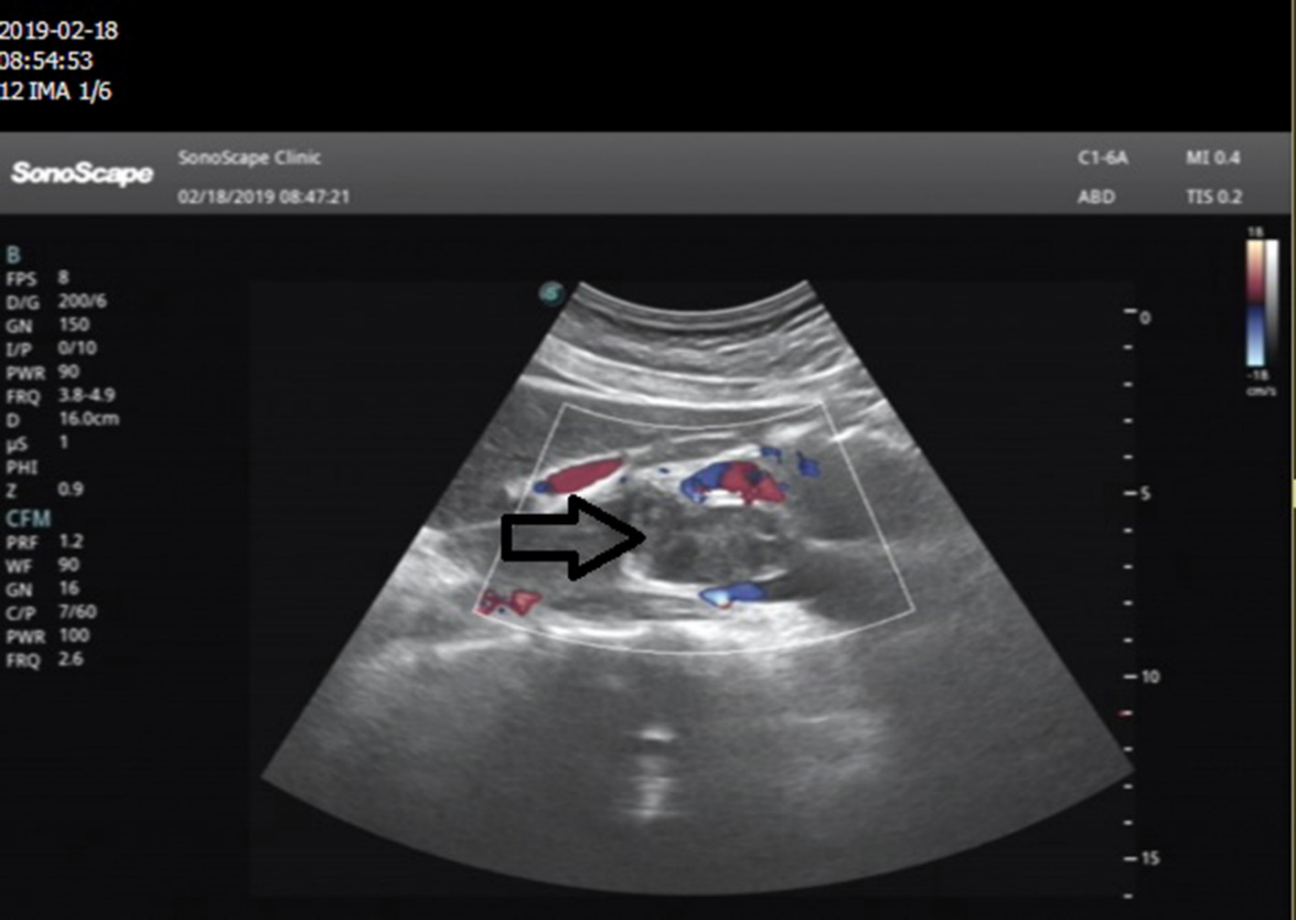
Abdominal ultrasonography images in the epigastric sagittal view: a hypoechoic, irregularly shaped solid mass was seen adjacent to the IVC.

**Figure 2:**
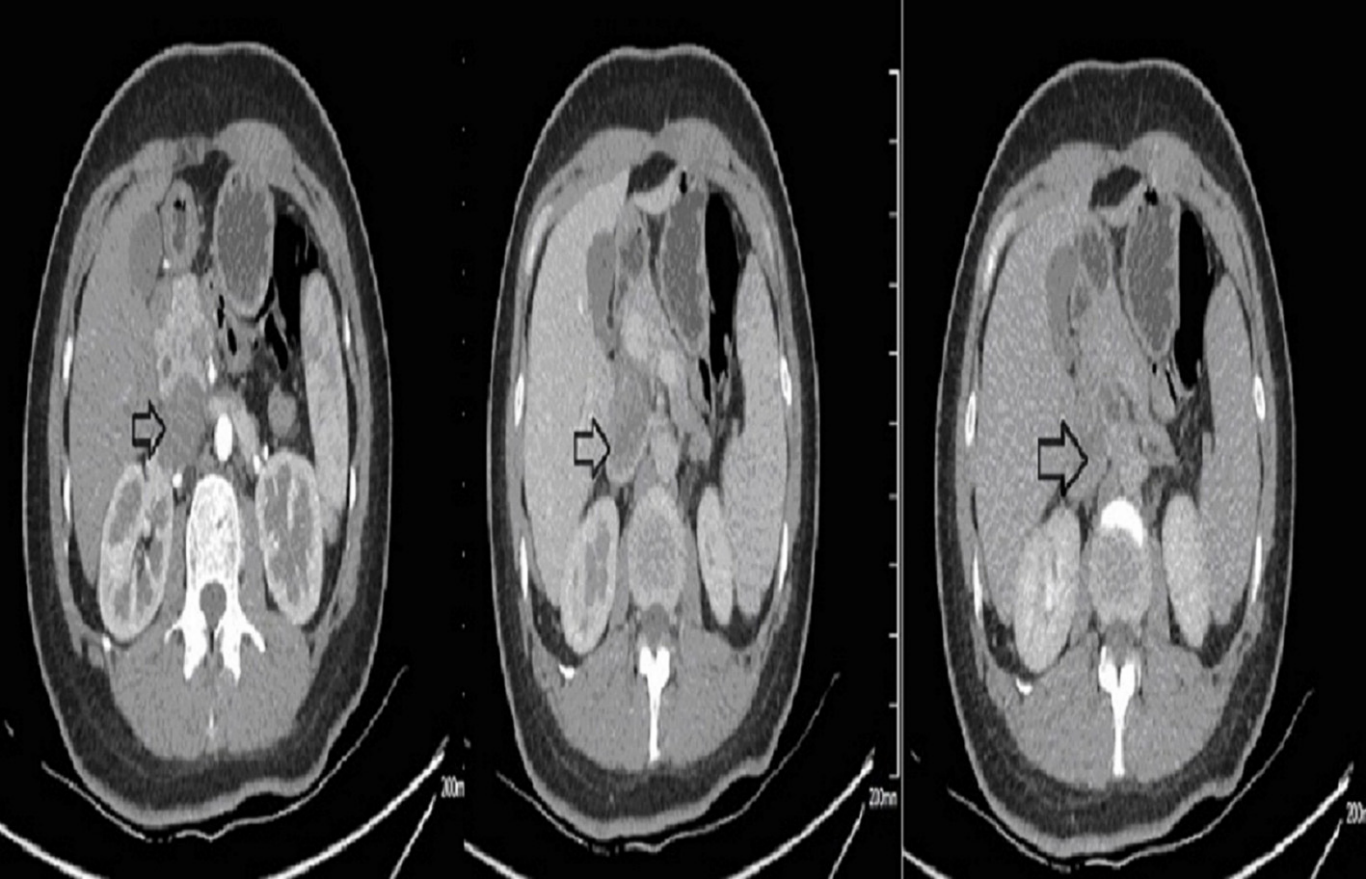
Preoperative contrast-enhanced CT images. The border between the tumor and IVC was indistinct.

**Figure 3:**
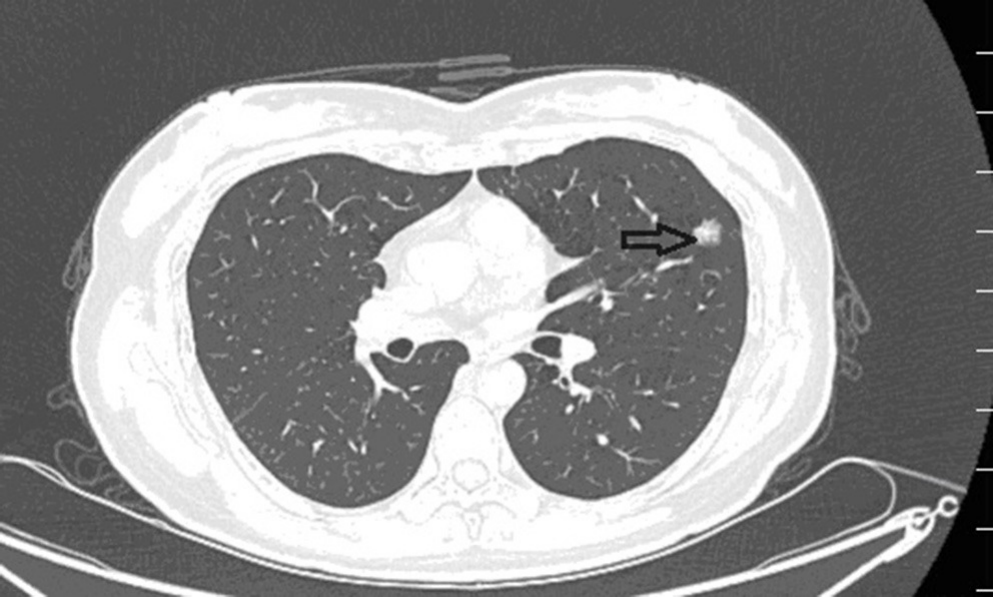
Preoperative contrast-enhanced CT images revealed a ball-shaped mass with heterogeneous enhancement in the left lung lobe.

Ultrasound of the abdomen showed a retroperitoneal tumor measuring 4.8 × 2.3 cm (Figure 1). To further confirm the diagnosis, Abdominal contrast enhanced computed tomography (CT) revealed a retroperitoneal mass 34 mm × 27 mm in size, near right renal veins extending to inferior vena cava, and it was pushing the pancreas forward (Figure 2). Abdominal contrast enhanced CT did not reveal any evidence of retroperitoneal enlarged lymph nodes. The border between the tumor and IVC was indistinct at the arterial phase, the venous phase and the delayed phase (Figure 2). To determine the primary site of the tumor and whether metastasis had occurred, The patients' complete examination included head magnetic resonance imaging (MRI), chest CT and cardiac ultrasound. The patient refused to undergo PET-CT due to financial constraints. The cardiac ultrasound and brain MRI were normal. The CT scan of the chest showed a tumor in the lingual segment of the upper lobe of the left lung, consisting of ground-glass nodules with unclear boundaries; The tumor was approximately 1.3 cm × 1.1 cm in size and multiple short burls can be seen around the edges of the tumor. There were no enlarged lymph nodes in the mediastinum (Figure 3). Bronchoscopy was performed to clarify the nature of the tumor, showing acute inflammation of the bronchial mucosa. Pulmonary tumor may be metastases of inferior vena cava tumors, primary lung cancer or benign pulmonary nodules. Therefore, the patient was discussed in the multidisciplinary tumor board meeting to determine the best treatment strategy. Discussion the results suggest surgery as the only effective treatment for inferior vena cava tumors and pulmonary tumor. The patient first underwent resection of IVC sarcoma. And 1 month later, the patient underwent wedge resection of pulmonary by thoracoscope without preoperative pulmonary biopsy.

The patient underwent resection of IVC sarcoma, which was located at the confluence of the renal vein into the inferior vena cava. After the proximal and distal clamp, the tumor and the involved IVC were completely removed from the infrahepatic IVC to just above the right renal vein. In order to save operation time, the inferior vena cava was reconstructed with artificial blood vessels. Blood loss was around 400 ml during the operation. Post operatively, patient made a good recovery without serious complications.

**Figure 4:**
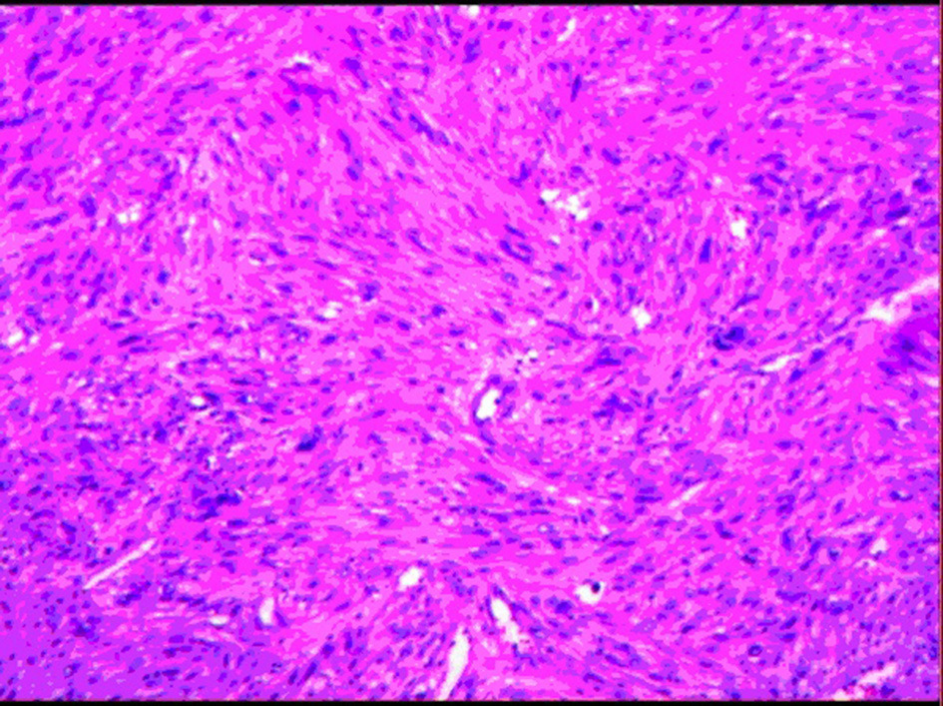
Image showing spindle-shaped tumor cells with areas of hypercellularity that were arranged in fascicles, bundles and interlacing patterns.

The postoperative pathological specimens showed that the size of the tumor was 6 × 4 × 3 cm, which was lobulated, the capsule was intact, and there was no metastasis in the surrounding lymph nodes. The histopathological examination showed a well-circumscribed mass composed of spindle cells arranged in interlaced and bundles (Figure 4). The tumor cells are fusiform, slender and rich in cytoplasm. The nucleus is deeply stained, the end is blunt, located in the center, with a certain degree of heteromorphism and pleomorphism (Figure 4). A few fused nuclei were also seen. Immunohistochemistry analysis revealed the tumor to be positive for smooth muscle actin (SMA), myoglobin, desmin, Epithelial Membrane Antigen (EMA), Transducin-Like Enhancer of split 1 (TLE-1), h-caldesmon, vimentin, CD99 and Ki-67:40%, and negative for CD 117, CKp, CK7, Bcl-2, S100, CD31, CD34, Dog-1 and STAT-6. These findings support the diagnosis of IVC sarcoma.

After 1 month, the patient underwent thoracoscopic wedge resection of the upper lobe of left lung. The lung tissues 20 mm from the edge of the nodule, including the nodule, was removed with a linear cutting closure device. There were no obvious blood loss and complications during or after the operation. Blood loss was around 50 ml during the operation. Post operatively, patient made a good recovery without serious complications.

**Figure 5:**
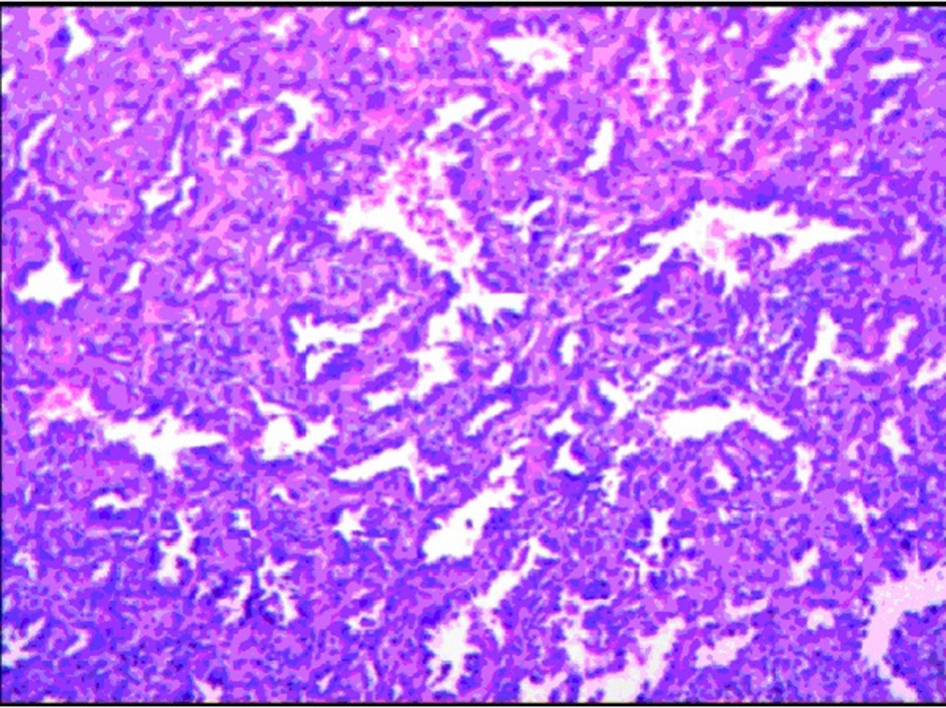
The histopathological examination of the excised mass combined with the pathology expert group discussion considered lung adenocarcinoma.

**Figure 6:**
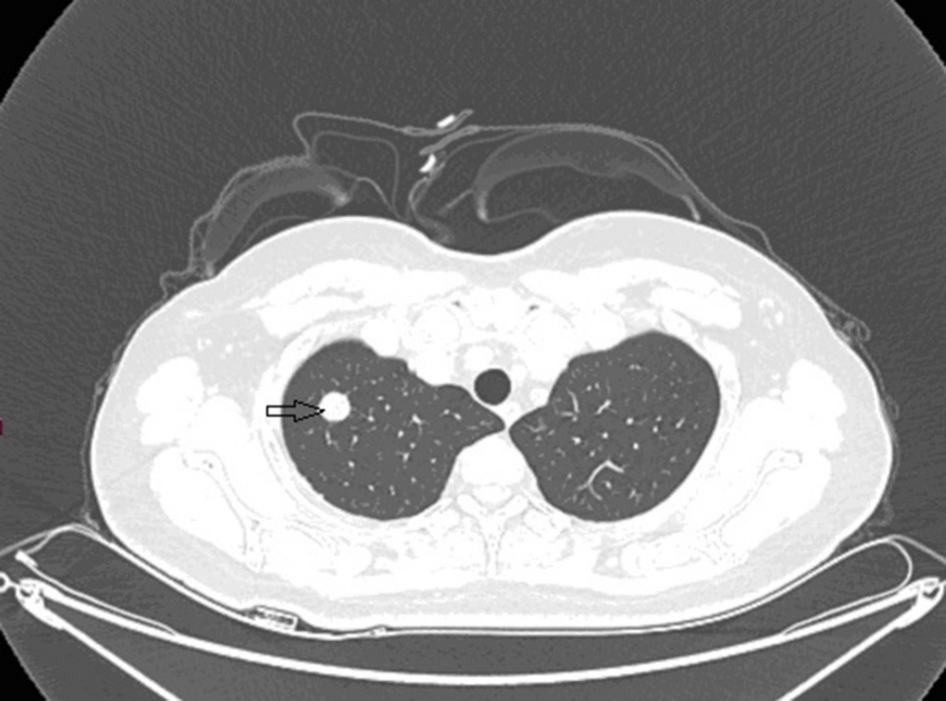
Contrast-enhanced CT images at 18 months after second surgery: lump in upper lobe of the right lung.

**Figure 7:**
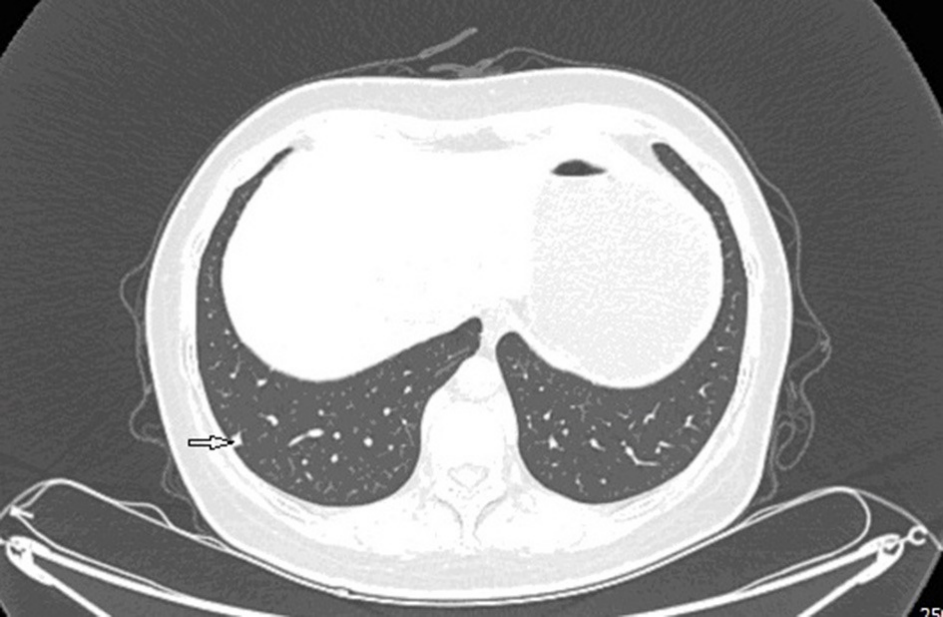
Contrast-enhanced CT images at 18 months after second surgery: lump in lower lobe of the right lung.

**Figure 8:**
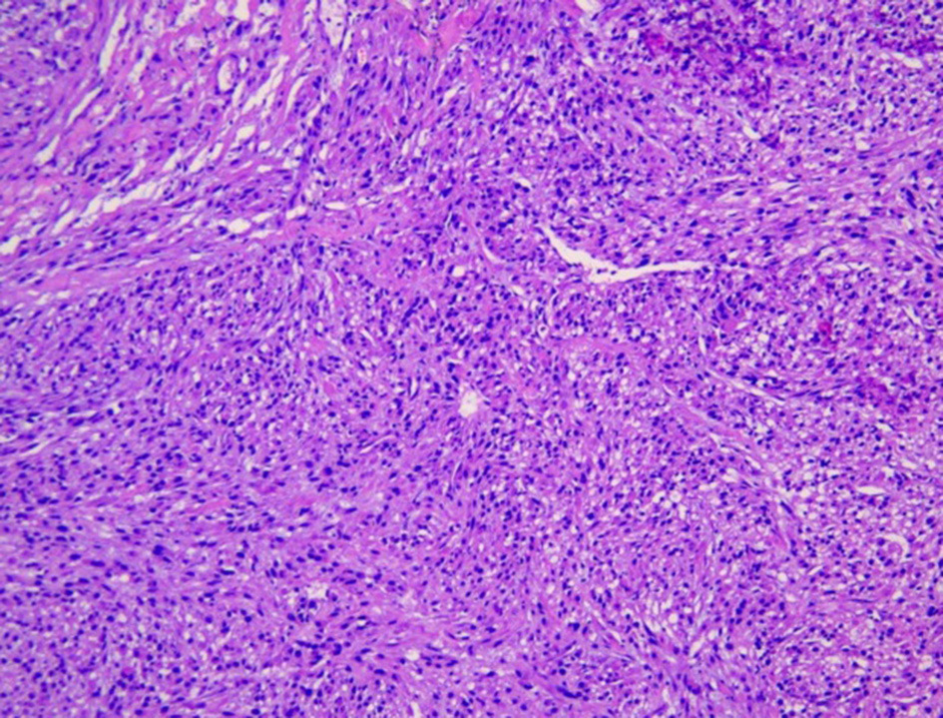
The metastasis of inferior vena cava sarcoma in lung.

**Figure 9:**
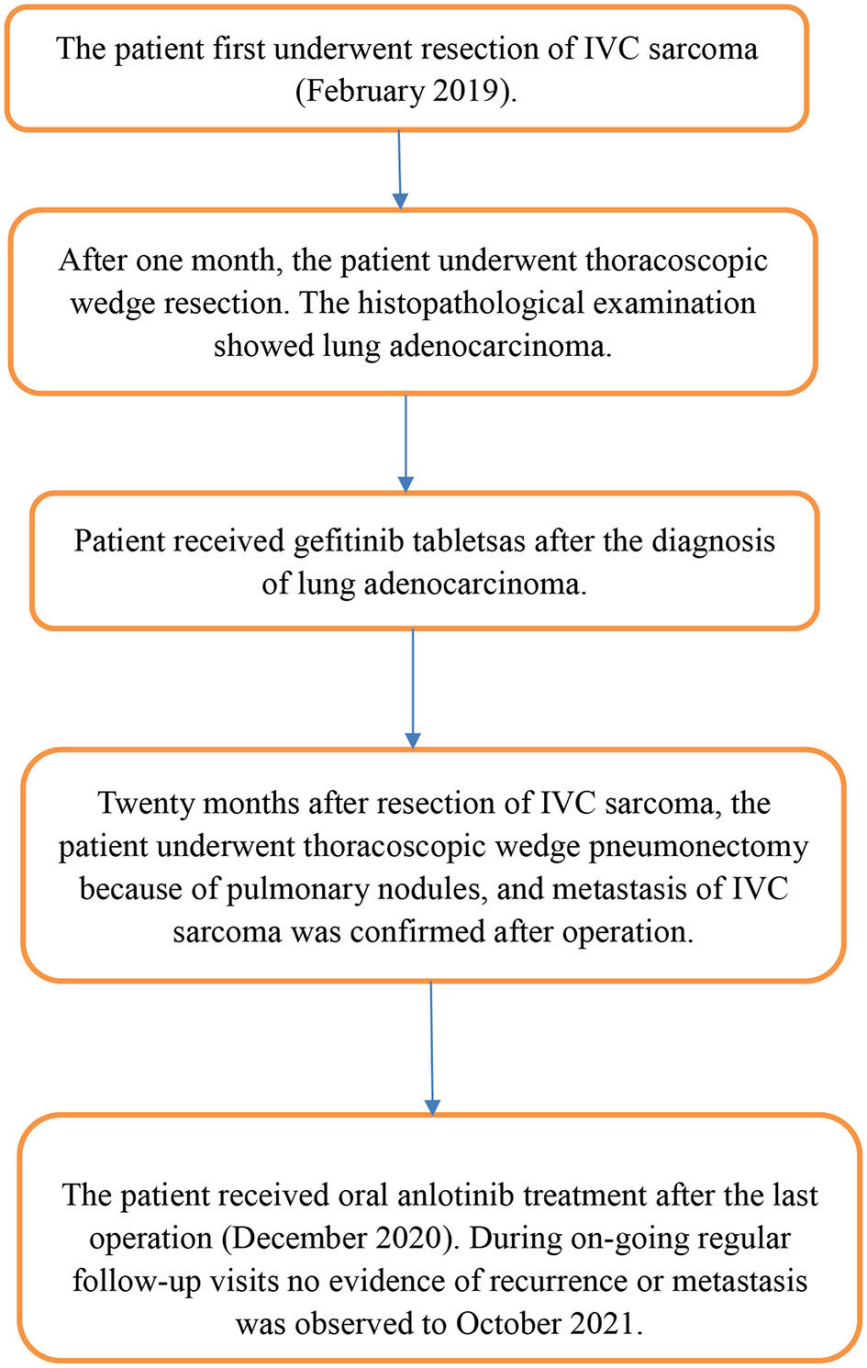
Historical and current information from this case organized as a timeline.

Grossly, the resected lung tissue was approximately 6 × 4 × 3 cm in size with an solid mass measuring 0.8 × 0.8 cm in the central area. Patient underwent pathologically confirmed complete anatomical resection of the primary tumor, therefore the surgical margin was >2 cm. There were no enlarged or suspected lymph nodes in the mediastinum during the operation, so mediastinal lymph node dissection was not performed. The histopathological examination of the excised mass showed lung adenocarcinoma (Figure 5). Immunohistochemical staining of the tumor cells revealed positive expression for CK7, napsin A, TTF-1 and Ki-67:20%, and negative expression for CK5/6, p63, p40, Syn, CgA, CD56 and LCA. Cells from a squamous cell carcinoma are generally CK5/6, p40 and P63 positive and TTF-1 and Napsin A negative whereas adenocarcinoma cells are generally CK7, Napsin A and TTF-1 positive while being negative for p40 and CK5/6 (6). In addition, the patient's lung tissue specimens were consulted by pathologists in Fudan University Cancer Hospital and Gansu Cancer Hospital, and the pathologists of the two hospitals agreed on the diagnosis of lung adenocarcinoma. The patient received gefitinib tablets (250 mg per day) as a maintenance therapy during the perioperative period. Follow-up based on patient's recent medical history, chest and abdominal physical examination, complete blood count, liver function tests, tumor markers monitoring, ultrasound scan of the abdomen every 3 months, CT scans of the lung, abdomen and head every 6 months. 20 months after resection of inferior vena cava leiomyosarcoma, the contrast-enhanced CT images show two lump about 1.2 and 0.7 cm in diameter in upper lobe (Figure 6) and lower lobe of the right lung (Figure 7). According to the examination results, it may be metastasis of inferior vena cava sarcoma or recurrence of lung adenocarcinoma. Thoracoscopic wedge resection was performed in patient after the discussion by thoracic surgery department. The immunohistochemical results of postoperative specimens were consistent with the metastasis of inferior vena cava leiomyosarcoma (Figure 8). Immunohistochemical staining of the tumor cells revealed positive expression for SMA, desmin, vimentin and h-caldesmon and Ki-67:70%, and negative expression for CD117, S100, CD34, and Dog-1. Histopathological examination of the right lung mass confirmed the diagnosis of the inferior vena cava leiomyosarcoma metastasis. The patient received oral anlotinib treatment (12 mg once daily) after the last operation. The follow-up items are the same as before. During on-going regular follow-up visits no evidence of recurrence or metastasis was observed from December 2020 to October 2021 (Figure 9).

## Discussion and Conclusions

Leiomyosarcomas, which are characterized by smooth muscle differentiation, can be found throughout the body where there is a vein (7). No more than 5% of leiomyosarcomas originate from large blood vessels, and most of these originate from the IVC (8). Leiomyosarcoma of the IVC was first described in 1871 (9). According to its location, the tumor is divided into three groups: segment I: infrarenal; segment II; inter-renal and supra-renal, up to but not including the main supra-hepatic veins; and segment III: supra-hepatic, up to the right atrium (10, 11). Because of the different dimensions, growth patterns, and locations of the tumor, leiomyosarcoma of the IVC has different symptoms. Patients present with right lower quadrant pain, back or flank pain, and leg oedema when the tumor is located in the infrarenal region, while renal involvement results in renal vein thrombosis, nephrotic syndrome or arterial hypertension. Patients with tumors in the upper portion of the IVC present with weight loss, nausea, Budd-Chiari syndrome or lower extremity oedema (10, 12).

Currently, a number of non-invasive imaging methods, including ultrasound, CT, and MRI, are available for the diagnosis of leiomyosarcoma of the IVC (13–15). Because of the lack of specificity, most of these techniques are considered non-diagnostic. The diagnosis of the IVC sarcoma is difficult, and due to the insidious course of the disease, and most cases are diagnosed at a late stage (16). The final diagnosis of the IVC leiomyosarcoma is based on pathology. SMA, myoglobin, and vimentin were positive for smooth muscle differentiation, and CD 117, Bcl-2, S100, CD31, CD34, Dog-1 and CK7 were negative for non-myogenic differentiation (17). Consequently, the immunohistochemical results of this study support the diagnosis of IVC sarcoma. Lung adenocarcinoma was diagnosed during evaluation of the IVC tumor, without any symptoms. Immunohistochemical staining of left lung tumor cells showed positive expression of CK7, napsinA and TTF-1. CK7, napsin A and TTF-1 have high specificity and sensitivity in the diagnosis of lung adenocarcinoma (18). In addition, in this case, the zone of transition from adenomatous to spindle cells could not be detected. Spindle cell tumors of the IVC were not derived from pulmonary adenocarcinoma. Thus, they could be two different types of primary tumors.

At present, radical surgical resection with free margins is believed to be the best curative therapy for the IVC leiomyosarcoma (14, 16, 19). The surgical approach differs depending on the tumor invasiveness and location, including simple tumor excision with IVC repair and tumor excision with graft replacement. The efficacy of chemotherapy and radiotherapy is limited (20). The prognosis of the IVC sarcoma depends on the tumor size and location as well as complete surgical resection (21). The 5-year survival rate following surgery has been reported to be ~50% (10, 22). Most leiomyosarcomas show a highly aggressive growth pattern with a high incidence of recurrence and metastasis. In this study, the patients were complicated with lung tumors and lacked more effective treatment. Therefore, in order to achieve the best therapeutic effect, we choose to remove the tumor in stages. Postoperative adjuvant therapy was given according to the results of the patient's pathological examination. The patient refused chemotherapy for IVC sarcoma and received targeted therapy for lung cancer after surgery. Oral anlotinib was started after the third operation when the patient was diagnosed with right lung metastasis from IVC sarcoma. A study showed that tumor recurrence occurs in more than half of these patients despite complete resection (10). So complete surgical resection alone is insufficient in achieving long-lasting tumor control.

To our knowledge, this is the first report the case of patient with a primary IVC leiomyosarcoma and lung adenocarcinoma. We did not find any similar cases in the literature. With that, surgery is still an effective treatment for patients with a primary IVC leiomyosarcoma and lung adenocarcinoma at present. This study also has shortcomings: on the one hand, the patients did not receive chemotherapy or targeted therapy for IVC sarcoma after the first surgery. On the other hand, patients who received anlotinib had a shorter follow-up period. In the future, due to the difficulty in early diagnosis in primary IVC leiomyosarcoma patients, there is an urgent need to develop new methods for the rapid diagnosis and treatment of this disease.

## Patient Perspective

I've had three surgeries. There is no obvious discomfort now. After the third operation confirmed pulmonary metastasis, there was occasional worry about the recurrence and metastasis of the disease. Now everything is normal in oral alotinib treatment.

## Data Availability Statement

The raw data supporting the conclusions of this article will be made available by the authors, without undue reservation.

## Ethics Statement

We confirm that manuscript containing any individual person's data received the consent of patients and their families for publication.

## Author Contributions

XG and ZW developed the idea of the case report and draft this manuscript. XG and MW contributed to the acquisition and interpretation of data. YZ provided critical review and substantially revised the manuscript. All authors read and approved the final manuscript and have contributed to and agreed on the content of the manuscript.

## Funding

This study was supported by the Cuiying Scientific and Technological Innovation Program of Lanzhou University Second Hospital (CY2018-MS14).

## Conflict of Interest

The authors declare that the research was conducted in the absence of any commercial or financial relationships that could be construed as a potential conflict of interest.

## Publisher's Note

All claims expressed in this article are solely those of the authors and do not necessarily represent those of their affiliated organizations, or those of the publisher, the editors and the reviewers. Any product that may be evaluated in this article, or claim that may be made by its manufacturer, is not guaranteed or endorsed by the publisher.

## Abbreviations

IVC: inferior vena cava
CT: computed tomography
MRI: magnetic resonance imaging
SMA: smooth muscle actin
EMA: Epithelial Membrane Antigen
TLE-1: Transducin-Like Enhancer of split.
